# Primary cerebral low-grade B-cell lymphoma, monoclonal immunoglobulin deposition disease, cerebral light chain deposition disease and “aggregoma”: an update on classification and diagnosis

**DOI:** 10.1186/1471-2377-13-107

**Published:** 2013-08-15

**Authors:** Marco Skardelly, Georgios Pantazis, Sotirios Bisdas, Guenther C Feigl, Martin U Schuhmann, Marcos S Tatagiba, Rainer Ritz

**Affiliations:** 1 Department of Neurosurgery, University Hospital Tuebingen, Hoppe-Seyler-Str. 3, 72076, Tuebingen, Baden-Wuerttemberg, Germany; 2 Institute of Brain Research, University Hospital Tuebingen, Hoppe-Seyler-Str. 3, 72076, Tuebingen, Baden-Wuerttemberg, Germany; 3 Department of Neuroradiology, University Hospital Tuebingen, Hoppe-Seyler-Str. 3, 72076, Tuebingen, Baden-Wuerttemberg, Germany

**Keywords:** Aggregoma, Light chain deposition disease, Lymphoma, Monoclonal immunoglobulin deposition disease, Neurooncology, Primary central nervous system lymphoma, Stereotaxic surgery

## Abstract

**Background:**

This work aims to add evidence and provide an update on the classification and diagnosis of monoclonal immunoglobulin deposition disease (MIDD) and primary central nervous system low-grade lymphomas. MIDD is characterized by the deposition of light and heavy chain proteins. Depending on the spatial arrangement of the secreted proteins, light chain-derived amyloidosis (AL) can be distinguished from non-amyloid light chain deposition disease (LCDD). We present a case of an extremely rare *tumoral* presentation of LCDD (aggregoma) and review the 3 previously published LCDD cases and discuss their presentation with respect to AL.

**Case presentation:**

A 61-year-old woman presented with a 3½-year history of neurologic symptoms due to a progressive white matter lesion of the left subcortical parieto-insular lobe and basal ganglia. 2 former stereotactic biopsies conducted at different hospitals revealed no evidence of malignancy or inflammation; thus, no therapy had been initiated. After performing physiological and functional magnetic resonance imaging (MRI), the tumor was removed under intraoperative monitoring at our department. Histological analysis revealed large amorphous deposits and small islands of lymphoid cells.

**Conclusion:**

LCCD is a very rare and obscure manifestation of primary central nervous system low-grade lymphomas that can be easily misdiagnosed by stereotactic biopsy sampling. If stereotactic biopsy does not reveal a definite result, a “*wait-and-see”* strategy can delay possible therapy for this disease. The impact of surgical removal, radiotherapy and chemotherapy in LCDD obviously remains controversial because of the low number of relevant cases.

## Background

Primary central nervous system lymphomas (PCNSL) are defined as non-Hodgkin’s (NHL) lymphomas that primarily arise in the central nervous system [1]. PCNSL account for approximately 1–2% of all primary cerebral tumors, and approximately 98–99% are classified as diffuse B-cell lymphoma (analogous to systemic B-cell NHL) [2,3]. Intracerebral manifestations of T-cell lymphomas and secondary lymphomas are extremely rare [3]. Low-grade PCNSL represents a less aggressive subgroup compared with systemic NHL and accounts for approximately 3–20% of all PCNSL [4,5]. Only a few low-grade PCNSL are associated with the deposition of monoclonal light and heavy chain immunoglobulins (Ig).

Monoclonal immunoglobulin deposition disease (MIDD) is characterized by the deposition of monotypic light and/or heavy chain proteins in various tissues and organs. MIDD mainly affects the kidneys, but the involvement of other organs (e.g., the liver, heart and peripheral nerves) is not uncommon [6]. All forms of MIDD can be ascribed to monoclonal expansion of an immunoglobulin (Ig) light and/or heavy chain producing B-cells [7]. 2 subgroups of MIDD can be differentiated histologically based on the different spatial arrangement of the secreted proteins. In the more common subgroup, the light chain-derived amyloidosis (AL) subgroup, proteins are aggregated in fibrils to ß-pleated sheets that stain for Congo red and display green birefringence under polarized light [8]. The second subgroup is characterized by ultrastructural non-organized proteins, which aggregate in more amorphous Congo red-negative depositions. Randall and colleagues initially described 2 patients with the systemic deposition of non-amyloid Ig light chains and proposed the term light chain deposition disease (LCDD) [9]. Subsequent reports confirmed the existence of systemic heavy chain deposition disease (HCDD) as well as both light and heavy chain deposition disease (LHCDD) [10].

We provide an update regarding the diagnosis and classification of primary cerebral low-grade B-cell lymphomas and cerebral light chain deposit diseases. We present the case of a patient with a 3½-year progressive hemiparesis and hemi-hypoesthesia of the right side due to a delayed diagnosis and therapy of the extremely rare, tumor-presenting cerebral restricted LCDD, which can be called “aggregoma” [11]. We further present a systematic overview and discussion of the disease with respect to light chain-derived amyloidosis.

## Case presentation

### Clinical presentation

**Figure 1:**
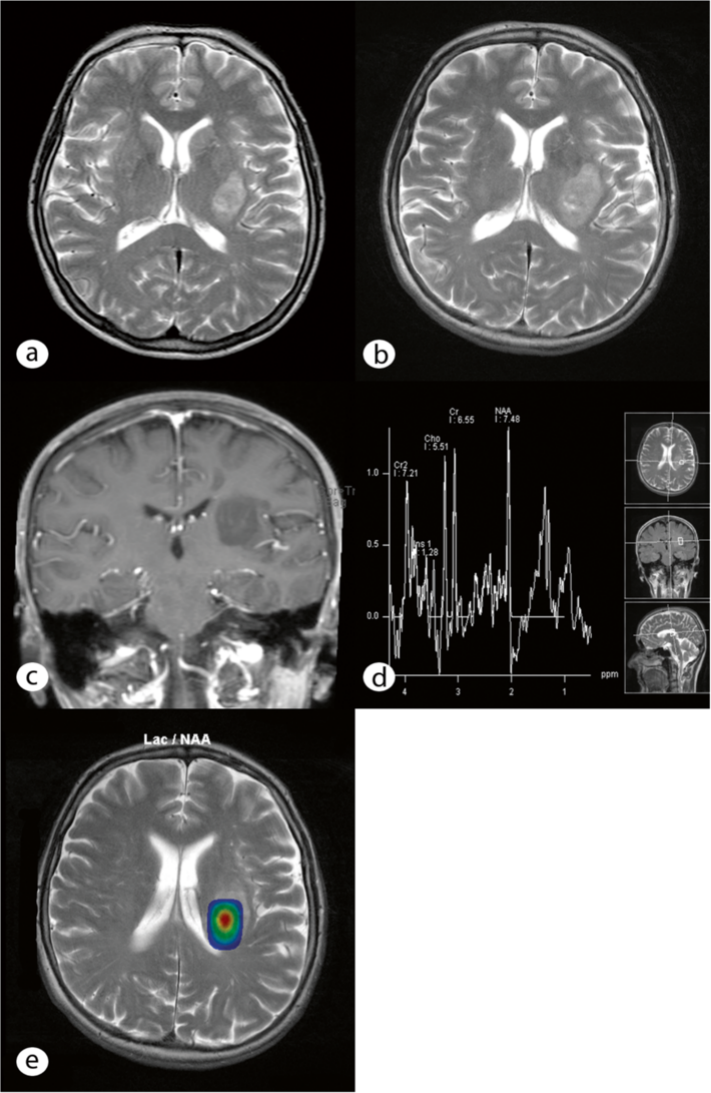
**MRI and MR spectroscopy of the tumor. (a)** Axial T2-weighted image shows a sharply demarcated, hyperintense lesion on the posterior aspect of the left basal ganglia and the subcortical left parieto-insular lobe. The lesion affects the internal and external capsules without reaching the insular cortex. **(b)** Follow-up MR imaging after 1 year demonstrates progression of the lesion size, particularly on the posterior part, with medially located hyperintense foci corresponding to microcysts on T2-weighted imaging. **(c)** Coronal T1-weighted MR imaging after gadolinium administration shows punctual enhancement within the lesion. **(d)** Proton MR spectroscopy using a short echo time (TE: 30 ms) on the lateral part of the lesion reveals a reduced NAA peak and reduced lactate levels (1.3 ppm). The lactate concentration appears more prominent on the medial part of the lesion (and correlates with the microcystic, necrotic changes observed in Figure 1b), as shown on the metabolic map overlaid on the axial T2-weighted image **(e)**.

A 61-year-old woman was admitted to our department with progressive brachiofacially accentuated hemiparesis, dysdiadochokinesia and hemi-hypoesthesia of the right side of the body, which began 3½ years previous. She initially presented with dyspraxia and fluctuating hypoesthesia of the right hand at the end of 2006; her cranial nerves were not affected. She complained of increased fatigue but did not present with weight loss, night sweats, fever or headache. The woman had a history of hypothyroidism related to Hashimoto’s thyroiditis and suffered endocarditis and streptococcal sepsis in 1982. She was under a long-term medication treatment of 100 μg of thyroxin daily. An MRI scan performed in May 2007 revealed a 2.8 × 2.0 × 2.4 cm lesion of the white matter at the level of the left subcortical parieto-insular lobe and basal ganglia (Figure 1a). The lesion presented as hypointense on T1-weighted scans with some regions displaying slight enhancement after gadolinium administration and moderate inhomogeneous hyperintensity on T2-weighted scans. Blood serum inflammatory markers (leukocytes and CRP), cerebrospinal fluid protein, and the cell count revealed no abnormalities. A stereotactic serial biopsy was performed in June 2007. The histological analysis demonstrated colloidal-bodied particles with scattered single cells with no proof of tumors or inflammatory cells. Based on the obvious diagnosis of an atypical colloidal cyst, no further therapy was initiated. The first control MRI (3 months after biopsy) demonstrated no progression of the disease. However, a clinical deterioration and progression of the lesion (3.6 × 2.3 × 2.6 cm) (Figure 1b &1c) at the follow-up MRI session in July 2008 warranted a stereotactic serial biopsy, which was conducted at a neurosurgical clinic that specialized in stereotactic procedures. Prior to the stereotactic surgery, CSI (chemical shift imaging) was performed, which revealed a prominently reduced NAA peak, an elevation of lactate and only slight alterations of choline and creatine peaks (Figure 1d &1e).

**Figure 2:**
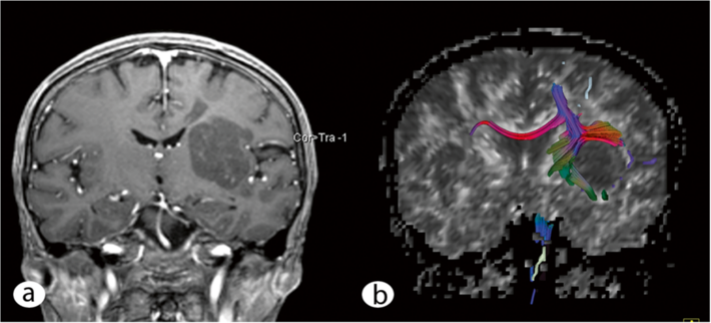
**Preoperative MRI and fiber tracking. (a)** Coronal T1-weighted image after gadolinium administration shows lesion progression. **(b)** The fiber tracking imaging based on the preoperatively performed diffusion tensor imaging demonstrates displacement of the corticospinal and corticopontine tracts on the left side, whereas the body of the corpus callosum seems unaffected.

The histological analysis of the biopsy that was performed in June 2007 showed a glial cyst but exhibited no evidence of malignancy or inflammation. In May 2010, the patient presented for the first time at our department because of clinical and radiological progression of the disease. Surgical resection of the lesion with a subsequent histological diagnosis was recommended At the day of admission, the patient displayed poorer health and a worsened nutritional status. The neurological examination demonstrated moderate hemiparesis of the right side and hemi-hypoesthesia, including the trigeminal nerve, with no other cranial nerve deficits. The woman had a high-grade disturbance of the fine motor skills of the right hand, dysdiadochokinesia of the right side and displayed an instable standing posture and gait. Her reflexes exhibited increased positive Troemner’s and Babinski’s signs. On the day of admission, a control MRI that included diffusion tensor imaging and a functional MRI were performed. The lesion progressed in volume to 4.2 × 3.2 × 5.3 cm (Figure 2a) with the same signal characteristics in T1- and T2-weighted imaging; displacement of the left pyramidal and corticopontine tract was observed (Figure 2b) with significantly altered values of the fractional anisotropy compared with the healthy contralateral side.

### Intervention and postoperative course

**Figure 3:**
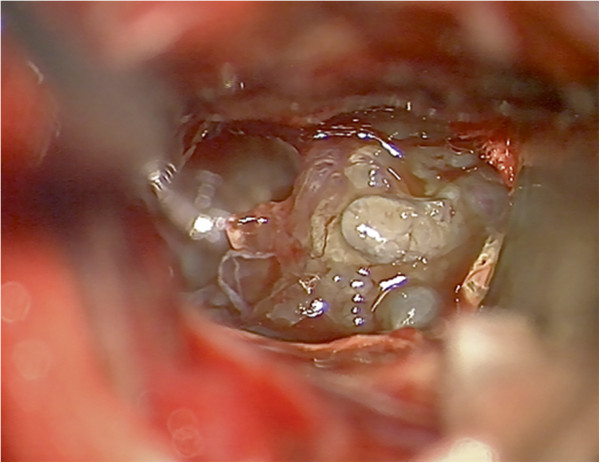
**Intraoperative microscopic image.** The image reveals the amorphous, gelatinous mass of the tumor.

The patient underwent a left-sided standard pterional craniotomy and a removal of the lesion via a transsylvian, transinsular approach (with respect to the displacement of the left pyramidal and corticopontine tracts) under continuous intraoperative monitoring with sensory evoked and motor evoked potentials (SEPs and MEPs, respectively). The lesion appeared intraoperatively as an amorphous, gelatinous mass with some regions appearing dull and lucid (Figure 3). The tumor mass could be almost completely removed with the preservation of the MEPs and SEPs. After the operation, the patient exhibited increased hemiparesis of the right side with hemiplegia of the right arm and partial facial palsy in addition to a new expressive aphasia. A postoperative head CT revealed no signs of bleeding or infarction. By the time of discharge, the patient had partially recovered from aphasia and was mobilized with a Zimmer frame. In the follow-up control in the outpatients’ department after rehabilitation in October 2010, the patient demonstrated almost complete recovery from expressive aphasia and had slight palsy of the marginal mandibular branch of the facial nerve but continued to display moderate brachially accentuated hemiparesis of the right side. Subsequently, the department of hematooncology initiated diagnostic staging, and no further manifestations of B-cell lymphoma could be identified. Whole-body CT, abdominal ultrasound and bone marrow puncture revealed no suspicious findings. Electrophoresis showed a regular distribution and quantity of serum proteins (7.6 g/dl) and immunoglobulins. Additionally, there was no hint of monoclonality of serum proteins in the immunofixation assay. Physiological albuminuria in urine electrophoresis was observed.

### Neuropathology

**Figure 4:**
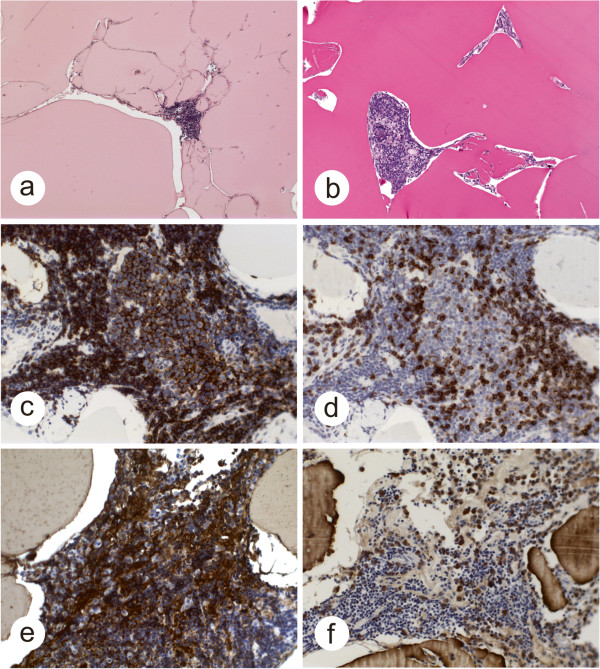
**Histological analysis of the tumor. (a)** Amorphous eosinophilic deposits (hematoxylin and eosin staining). **(b)** Amorphous deposits are strongly PAS positive. **(c)** Most of the lymphoid cells express CD20. d) Few infiltrating CD3 positive T-cells are observed. Amorphous deposits and plasmacytic cells stain positive for Ig-A heavy chain **(e)** and for λ-light chain antibody **(f)**.

Histological examination of the resected tumor revealed large, amorphous, proteinaceous eosinophilic deposits and small islands of lymphoid cells between them (Figure 4a). The amorphous deposits were strongly PAS positive but negative for Congo red staining and did not display apple-green birefringence under polarization (Figure 4b). Most of the small lymphoid cells expressed the B-cell marker CD20 (Figure 4c). There were also few CD3-positive T-cell lymphocytes (Figure 4d). Immunoreactivities for the Ig-A heavy chain (Figure 4e) and λ-light chain immunoglobulins (Figure 4f) were partially observed in the deposits, plasmacytoid lymphocytes and mature plasma cells. The plasmacytic cells expressed CD38 and CD138, whereas they were negative for C79a. There was a negative reaction for IgM, IgG and IgE heavy chains and for κ-light chains. The lymphoid cells had a low proliferation rate (IHC for Ki67, 3 %). Some expressed bcl2, a few lymphocytes expressed CD5, and there was negative immunoreactivity for CD10, CD19, MUM1 and bcl6. Semi-nested PCR revealed monoclonal amplification of the complementarity-determining region III (CDR III) of the immunoglobulin heavy chain gene [1], supporting the diagnosis of low-grade lymphoplasmacytic lymphoma with light chain deposition disease and IgA- and λ-light chain restriction.

## Discussion

Insoluble amyloid- or light chain-related non-amyloid aggregates can be formed by systemically secreted proteins, mainly involving the kidneys or occurring in an organ-specific manner [12]. Different CNS diseases are associated with amyloid depositions in the brain (e.g., Alzheimer’s disease, cerebral amyloid angiopathy, prion-related diseases or light chain-derived amyloidosis (AL)) [13]. The organ-specific, space-occupying solitary tumoral presentation of AL is called *amyloidoma*; regarding LCDD, the corresponding term *aggregoma* was suggested by Rostagno et al. in 2002. He first reported 3 patients who had tumoral masses of non-amyloid κ-light chain aggregates: 2 with cervical masses and 1 with a solitary lung nodule [11]. To date, 50 cases of cerebral-restricted AL have been reported [14-16]. In addition to 30 intracerebral solitary amyloidomas that mainly exhibited λ-type amyloids (but in some cases both λ- and κ-type amyloids), 20 further cases that exclusively demonstrated λ-type amyloids were published [14,16]. The latter included 8 cases of restricted AL with intracranial plasmocytomas; 2 cases with PCNSL; 1 case with leptomeningeal amyloid angiopathy; 6 cases with multiple sclerosis; and 3 cases with widespread subcortical vascular amyloidosis with leukoencephalopathy (WSVAL). For further details, see the review by Schröder and colleagues [15]. By contrast, excluding our case, only 3 intracerebral LCDD cases were previously reported: 2 diffuse manifestations of λ-light chain aggregates [17,18] and 1 case of vascular presentation of non-amyloid λ-light chain aggregates, designated as cerebral LCDD vasculopathy (CLCDDV) [19]. For details about these cases see “Additional file 1”.

**Figure 5:**
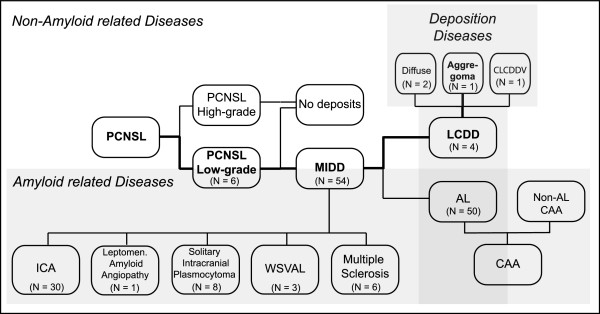
**Schematic of classification of the disease.** Pathological presentation of both entities of monoclonal immunoglobulin deposition diseases (MIDD), light chain-derived amyloidosis (AL) and light chain deposition disease (LCDD) in relation to the underlying disorders. The assignment of the reported case within the pedigree is highlighted in bold letters and lines. CAA = Cerebral amyloid angiopathy; CLCDDV = Cerebral LCDD vasculopathy; PCNSL = Primary central nervous system lymphoma; ICA = Intracerebral amyloidoma, WSVAL = Widespread subcortical vascular amyloidosis with leukoencephalopathy.

The case presented here is the first report on tumoral presentation of a brain-restricted LCDD that can be called an *aggregoma*. To create a better understanding of the relationships of the involved diseases they were classified in a pedigree (Figure 5); an overview of the details of the different manifestations of MIDD, AL and LCDD is provided in Table 1.

**Table 1:** Summary of findings of intracerebral light chain deposition diseases (No. 1–4) in comparison to intracerebral amyloidomas (No.5)

**No. and Source**	**1**	**2**	**3**	**4**	5*
	**Fischer et al. **[18]**2006**	**Popovic et al. **[19]**2007**	**Pantazis et al. **[17]**2010**	**Present case**	**Foreid et al. **[16]**2010**
					**Fischer et al. **[14]**2007 - (1935–2010) -**
**Age**	19	35	72	61	49 (15–71)
**Sex**	M	M	M	F	13F, 17M
**Initial clinical presentation**	Generalized epileptic seizure	A) Progressive	Focal epileptic seizure (hemiparesis)	Hemiparesis	Epileptic seizures (12)
		bulbar palsy			
					Hemiparesis (6)
		B) Putative			
					Gait disturbance (2)
		Paranoid			Visual impairment (4)
		schizophrenia			
					Cognitive disturbance (5)
					Hearing loss (1)
**Duration of symptoms**	No	A) 1 ½ years	8 years	3 ½ years	NA
		B) 13 years			
**First location**	Subcortical	Subcortical	Subcortical	Subcortical parieto-insular lobe & basal ganglia	Subcortical (21)
	periventricular posterior horn	gyrus rectus &	periventricular parietal lobe		Cortical (1)
		periventricular			
					Subcortical & cortical (8)
**Diagnostic**	Stereotactic Biopsy	Autopsy	Stereotactic Biopsy	Resection	Resection (9)
					Free-hand Biopsy (3)
					Stereotactic Biopsy (13)
					Autopsy (5)
**CT and MRI-Findings**	T2: Diffuse hyperintense mass with perifocal edema	NA in detail	T1 + GL:	T1: Hypointense	CT: Hyperdense
			Not enhanced	T1+GL: Enhanced	CT+CM: Enhanced
			T2: Hyperintense		T1: Hypointense
				T2: Iso- to	T1+GL: Enhanced
				hyperintense	T2: Iso- to
					hyperintense
**Histomorphology**	- Light chain deposits within vessel wall	- Light chain deposits	- Light chain deposits around blood vessels	- Light chain deposits	- Amyloid deposits
	- B cell lymphoma with plasma-cellular different	- around blood vessels	- B cell lymphoma with abundant plasma cells	- Diffuse lympho- plasmacytic B-cell lymphoma	- around blood vessels
	- Infiltrating T-cells	- within vessel wall	- Infiltrating T-cells	Infiltrating T-cells	- within vessel wall
		- Monoclonal B- cell proliferation			- In most cases NA, but in some cases lymphocytes and plasma cells
		- Infiltrating T-cells			
**Immunohistochemistry**	- Congo red neg.	- Congo red neg.	- Congo red neg.	- Congo red neg.	- Congo red positive
	- λ-Light chain deposits	- λ-Light chain deposits	- λ-Light chain deposits	- λ-Light chain deposits and IgA	- λ-Light chain deposits
	- Plasma cells: λ-light chain >> κ-light chain	- Plasma cells: λ-light chain >> κ-light chain	- Ki67: 2%	- Ki67: 3%	- 7–12-nm fibrils
	- Ki67: 1-3%	- CD3^+^, CD5^+^, CD20^+^, CD23^-^, Cyclin D^-^	- CD5^+^, CD38^+^, CD19^+^, CD10, CD79a^-^, CD138^-^	- CD20^+^, CD5^+^, CD3^+^ CD38^+^, CD19^-^, CD10^-^, MUM1^-^, bcl6^-^	- λ-light chain ++ 14/16
					- κ-light chain + 2/16

* N = X/30, number of patients with the presented characteristics of all presented cases of all N=30 intracerebral amyloidomas.

In systemic amyloidosis or LCDD, the CNS is usually not affected because of the blood–brain-barrier (BBB), which protects the brain sufficiently from circulating harmful metabolites (e.g., misfolded proteins) [20]. Nevertheless, 54 cases of either brain-restricted amyloid or non-amyloid deposition diseases, including our case, have been reported thus far (Table 1). Most of the depositions were located in the subcortical periventricular white matter, a preferred location for B-cell lymphoma [5]. Plasma cells were associated with deposits in many of the published cases with proof of monoclonality in some cases, suggesting isolated intracerebral production of monoclonal immunoglobulins by monoclonal B-cell lymphocytes [15,19,21]. It appears that the BBB exerts its function of controlling the exchange of metabolites in both directions because in none of the 54 cases were other organ systems involved. In both isolated cerebral LCDD and AL, it has been shown that brain deposits mainly consist of λ-type light chains that form either amyloid or non-amyloid aggregations. Only in 5 cases of ICA were both κ- and λ-type amyloids demonstrated [16-19]. It is not clear why the same proteins demonstrate different behaviors of aggregation; however, there is evidence that both the micro-environmental conditions and intrinsic amino acid sequence determine protein aggregation behavior [18]. To date, all 3 published *aggregoma* studies revealed κ-light chains, exclusively [11]. Hence, under micro-environmental conditions that are not associated with the formation of amyloids, tumoral aggregation of light chains could be restricted to the sequence of κ-light chain amino acids. However, we provide evidence of a tumoral formation of λ-light chain deposits, which confounds this notion.

As in other neurological diseases, the clinical presentation of MIDD depends mainly on the location of protein deposition and not on the histological finding. The major clinical signs of LCDD in the brain are epileptic seizures, cognitive impairments, headaches, and in the case presented here, hemiparesis, all of which were the main neurological symptoms observed in intracerebral amyloidoma (Table 1; for details, see Additional file 1). Cerebral imaging techniques such as CT and MRI are rarely specific for the diagnosis of the underlying histopathology. In the case presented here, we observed a tumor mass that was hypointense in T1-weighted scans and isointense to hyperintense in T2-weighted scans with a slight enhancement after an application of gadolinium. These MRI characteristics are also usually observed in ICA (Table 1; for details, see Additional file 1). Other published cases of brain-isolated LCDD showed similar MRI properties but a more diffuse protein deposition similar to other brain diseases such as low-grade astrocytomas, cerebral lymphomas and inflammatory diseases of the white matter.

Because both, the clinical presentation and imaging features of various cerebral disorders are not specific, histological analysis functions as a pivot point for further therapeutic strategy. Depending on the location of the disease, the distinction of adjacent healthy brain tissue and the involvement of eloquent areas, a straightforward surgical removal, or an open alternative stereotactical biopsy would be the methods of choice. For ICA, it is known that medical treatment is not successful, but surgical removal has a good prognosis if the tumor can be removed completely [22,23]. For cerebral-restricted LCDD, only data from the therapy of 2 cases are available thus far and only for a time period of 24 months at the longest. Fischer et al. reported that after 3 cycles of methotrexate, the disease was steady for at least 24 months after the onset of symptoms and even a slight decrease in lesion size was observed by MRI after 6 months (for details, see Additional file 1*, Case I*). Although Pantazis and colleagues also demonstrated stability of the disease by 20 months after chemotherapy with Rituximab (MabThera™, Rituxan™), Trofosfamide (Ixoten™) and steroids, the control MRI new lesions after 16 months (for details, see Additional file 1*, Case III*). The data suggest that chemotherapy can provide at least a temporary benefit for disease progression but is most likely not sufficient to cure the disease possibly because of the drugs that cannot enter the brain across the intact BBB in sufficient levels to exert their full effect. One would assume an even weaker effect on *aggregomas* because penetration of the drugs across the BBB into the space-occupying lesion is strongly impaired, as it was also demonstrated for ICA [22].

## Conclusion

MIDD is a very rare and obscure manifestation of primary central nervous system low-grade lymphoma. The disease is characterized by monoclonal production of immunoglobulins that can occur in an isolated manner in the brain due to the BBB. The case on tumoral aggregation of λ-light chain deposits presented here, confounds the former assumption that tumoral aggregation of light chains is restricted to κ-light chain amino acids. To date, only 53 cases of MIDD have been published, including 50 cases of cerebral-restricted light chain-derived amyloidosis and 3 cases of the even less frequent non-amyloid light chain deposition disease. In the case presented here, the lesion was located in an eloquent area of the brain, which necessitated 2 stereotactic biopsies, both of which were performed at other hospitals (one in June 2007 and the other in July 2008) and demonstrated no evidence of malignancy or inflammation. However, the patient showed continuous tumor progression and clinical deterioration. After resection, histopathological examination determined that most of the tumor consisted of large, amorphous, proteinaceous, eosinophilic deposits with small islands of lymphoid cells between the deposits. The lack of awareness of LCDD and its unique histological presentation contribute to the risk of misdiagnosis and underscore the need for a careful description of this disease and the limitations of biopsy. To date, the best therapeutic strategy for this disease remains controversial because the impact of surgical removal, radiotherapy and chemotherapy cannot be reliably evaluated due to the low number of cases thus far.

## Consent

“Written informed consent was obtained from the patient for publication of this case report and any accompanying images. A copy of the written consent is available for review by the Series Editor of this journal”.

## Competing interests

The authors declare that they have no competing interests.

## Authors’ contributions

MS composed the abstract, introduction and discussion, as well as the conclusion of the manuscript and created the illustration of the involved diseases. GP did the histological analysis, created the histological figures and wrote the histological section. SB created the MRI figures, did the MRI image analysis and wrote the MRI section. GF treated the patient on the ward, helped with the draft of the clinical case and performed the literature search. MS performed the surgical intervention, prepared the intraoperative picture and wrote the surgical section. MT did the surgical intervention, initiated the draft of the case report and helped with the design of the manuscript. RR participated in the design of the case report and coordinated and substantially helped with the draft of the key manuscript sections. All authors read and approved the final manuscript.

## Pre-publication history

The pre-publication history for this paper can be accessed here:

http://www.biomedcentral.com/1471-2377/13/107/prepub

## Supplementary Material

Additional file 1 LCDD - case summary. Click here for file
