# Supplementary material for: Primary cerebral low-grade B-cell lymphoma, monoclonal immunoglobulin deposition disease, cerebral light chain deposition disease and “aggregoma”: an update on classification and diagnosis
Source: BMC Neurol. 2013 Aug 15;13:107. doi: 10.1186/1471-2377-13-107 (PMC3751626; doi:10.1186/1471-2377-13-107)
Supplement: Additional file 1 — LCDD - case summary. [file 1471-2377-13-107-S1.doc]

**Additional file 1**

**Case I**

Fischer and colleagues presented a 19-year-old previously healthy man who was admitted to the hospital unconscious with a deviated gaze to the right [18]. The patient demonstrated repetitive, generalized seizures after intubation and exhibited a lesion that was hyperintense in T2-weighted MRI in the white matter adjacent to the right posterior horn. A stereotactic biopsy revealed B-cell lymphoma with plasmacellular differentiation and λ-light chain deposits. PCR analysis of the CDR III region exhibited only polyclonal Ig heavy chain rearrangements. Cerebrospinal fluid (CSF) investigation showed only a slight elevation of proteins (0.62 g/l), a normal cell count (<5 cells/µl) and no oligoclonal bands. Systemic manifestation of B-cell lymphoma was excluded by CT scans, clinical examination, blood and urine analysis and bone marrow biopsy. The patient received 3 cycles of chemotherapy with methotrexate. MRI follow-up demonstrated a slight decrease in lesion size after 3 and 6 months, and the clinical situation remained stable for at least 24 months.

**Case II**

This 35-year-old man died approximately 1½ years after the onset of neurological disorders and 13 years after onset of psychiatric symptoms [19]. He had been admitted to the hospital 7 times in 12 years because of varying psychiatric symptoms. At the age of 34 years, he developed cheek and neck stiffness and a slightly lowered right mouth angle. Because of aspiration, he had been transferred to the intensive care unit. MRI revealed multiple periventricular lesions. In the following month, the patient developed progressive cognitive impairment, dysarthria, impulsiveness, and subsequently, dysphagia and weakness of the upper extremities. Shortly afterward and without receiving specified treatment, he died. Autopsy revealed multiple intracerebral vessels that demonstrated narrowing in some parts with obliterated walls due to an overload of Congo red-negative extracellular, amorphous deposits, which were positive for λ-light chains. These vessels were mainly located in the subependymal white matter (except in the cortex) but also massively affected the medulla oblongata. Between vessels and deposits, mononuclear cells, mostly B-lymphocytes and plasma cells, could be detected. PCR revealed monoclonal rearrangement of the heavy chain Ig gene. CSF examination showed a normal protein concentration (0.36 g/l), slightly elevated cell numbers (7 cells/µl) and oligoclonal bands that were negative in serum, leading to the suspected diagnosis of multiple sclerosis. No further organs were affected.

**Case III**

Pantazis et al. presented a case of a 72-year-old man with residual hemiparesis of the left side due to a suspected stroke 8 years previously, after which the patient developed a focal motor seizure [17]. The initial MRI demonstrated slight T2-weighted hyperintense signal changes in the periventricular region and in the white matter of the parietal lobe. The follow-up MRI revealed multiple T2-weighted hyperintense white matter lesions of the complete right parietal and occipital lobes. CSF analysis showed a slightly increased albumin concentration (0.4 g/l), a borderline cell count (5 cells/µl) but a more than tenfold intrathecal synthesis of IgG (IgG 445 mg/l; normal range: 5–36) with a normal serum IgG level. A stereotactic biopsy revealed multiple perivascular Congo red-negative λ-light chain deposits in the white matter. In close vicinity of the vessels, lymphocytic infiltrates consisted of a few T cells; many plasma cells and B-cells were also detected. The suspected diagnosis of low-grade lymphoplasmacytic B-cell lymphoma could be confirmed by PCR analysis. Systemic involvement was excluded. Under immunosuppressive therapy with Rituximab (MabThera®, Rituxan®), Trofosfamide (Ixoten®) and steroids, the patient showed no clinical deterioration for at least 20 months. The follow-up MRI 16 months after the initiation of immunotherapy revealed a regression of the blood-brain-barrier disruption in the contrast-enhanced T1-weighted images. Nevertheless, disease progression with new hyperintensities in the white matter of the right frontal lobe was also observed.
